# Health, and How to Promote It

**Published:** 1879-05

**Authors:** 


Health, and How to Promote It. By Richard McSherry, M.D., Professor in Medical University of Maryland, etc. D. Appleton & Co. 549 & 551 Broadway, New York. 1879.

We have hardly had time to review in detail this little volume of 185 pages, but upon a superficial examination are satisfied that the important subject of hygiene will find a good auxiliary in the suggestions of this work. The total experience of the last few years has demonstrated the necessity of a perfected system of hygenic laws and regulations. Upon the establishment of these and their rigid execution much devastation and suffering will be prevented. The discussion of this suject, as presented in the above book, will recommend it to those feeling an interest in the question.

